# Light-driven biocatalytic reduction of α,β-unsaturated compounds by ene reductases employing transition metal complexes as photosensitizers†
†Electronic supplementary information (ESI) available: Additional experimental sections, tables, figures and discussion. See DOI: 10.1039/c5cy01642h



**DOI:** 10.1039/c5cy01642h

**Published:** 2015-10-26

**Authors:** Martyn K. Peers, Helen S. Toogood, Derren J. Heyes, David Mansell, Benjamin J. Coe, Nigel S. Scrutton

**Affiliations:** a Manchester Institute of Biotechnology , Faculty of Life Sciences , University of Manchester , 131 Princess Street , Manchester , M1 7DN , UK . Email: nigel.scrutton@manchester.ac.uk; b School of Chemistry , University of Manchester , Oxford Road , Manchester , M13 9PL , UK

## Abstract

Efficient and cost effective nicotinamide cofactor regeneration is essential for industrial-scale bio-hydrogenations employing flavin-containing biocatalysts such as the Old Yellow Enzymes. A direct flavin regeneration system using visible light to initiate a photoredox cycle and drive biocatalysis is described, and shown to be effective in driving biocatalytic activated alkene reduction. Using Ru(ii) or Ir(iii) complexes as photosensitizers, coupled with an electron transfer mediator (methyl viologen) and sacrificial electron donor (triethanolamine) drives catalytic turnover of two Old Yellow Enzymes with multiple oxidative substrates. Therefore, there is great potential in the development of light-driven biocatalytic systems, providing an alternative to the reliance on enzyme-based cofactor regeneration systems.

## Abstract 2

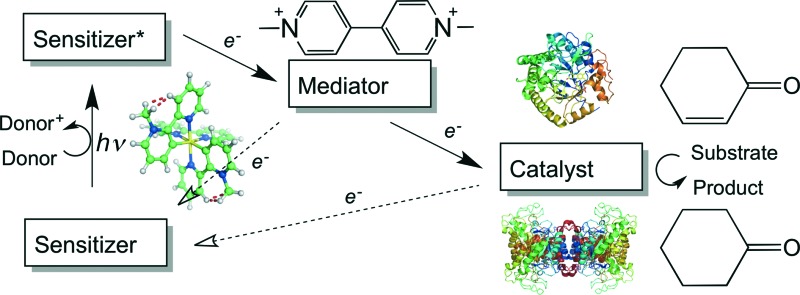
Efficient and cost effective nicotinamide cofactor regeneration is essential for industrial-scale bio-hydrogenations employing flavin-containing biocatalysts such as the Old Yellow Enzymes.

## 1 Introduction

Biocatalysis is an attractive alternative to more traditional chemical transformations, potentially offering superior regio-, stereo- and enantioselectivity to current synthetic procedures. A well-studied class of biocatalysts is the FMN-containing Old Yellow Enzyme (OYE) family of oxidoreductases.^1–3^ These nicotinamide adenine dinucleotide phosphate (NADPH)-dependent enzymes catalyse the hydrogenation of a variety of industrially-relevant α,β-unsaturated ketones, aldehydes and nitroalkenes. Due to the requirement for stoichiometric quantities of costly redox coenzymes, the development of efficient methods to facilitate *in situ* reduced FMN cofactor regeneration is critical for commercial viability. Existing methods include the addition of an enzyme-coupled NADP^+^/NADPH recycling system, requiring a sacrificial substrate to drive the process. Examples include dehydrogenases acting on alcohols, glucose, phosphite and formate.^4–8^ While efficient, this approach requires the maintenance of two enzymatic processes, adding to the complexity of the reaction. Chemical regeneration of NADPH utilising a variety of transition metal complex catalysts has been demonstrated, but poor aqueous solubility and reagent cost have prevented a more general use of this approach.^9–12^ Additionally the disproportionation reaction of conjugated enones, coupled with *in situ* product removal using a polymeric adsorbent MR-carbonate, has been developed as a ‘coupled substrate’ approach for OYE-catalysed reduction of activated alkenes.^12^ The use of inexpensive biomimetic analogues of nicotinamide coenzymes has also been described recently in OYE-driven catalytic hydrogenations.^13^

A variety of photosensitizers encompassing organic compounds,^15–21^ quantum dots^22^ and coordination complexes^14,23–28^ has been investigated in conjunction with enzymatic systems, for both mechanistic studies of electron transfer and studies of biocatalytic turnover. The most widely investigated photosensitizers are compounds of ruthenium(ii), more specifically those based upon the cation tris(2,2′-bipyridyl)ruthenium(ii), [Ru(bpy)_3_]^2+^, and analogous polypyridyl complexes. These compounds are ideal candidates as photosensitizers owing to a fortuitous combination of chemical stability, redox activity and long-lived excited states.^29^

A range of electrochemical techniques has been developed using direct, indirect or enzyme-coupled methodologies.^30–32^ Unfortunately, such methods often suffer from low efficiencies due to unwanted side-reactions, and require the use of specialized equipment with limited scale-up potential. An alternative direct reduced FMN regeneration system, employing visible light as a means of initiating a photoredox cycle to drive biocatalytic activity, offers a more general approach and has not been explored in relation to OYE biocatalysis. This approach would require minimal specialist knowledge or equipment and could be readily implemented at both the research and industrial scale.

**Scheme 1:**
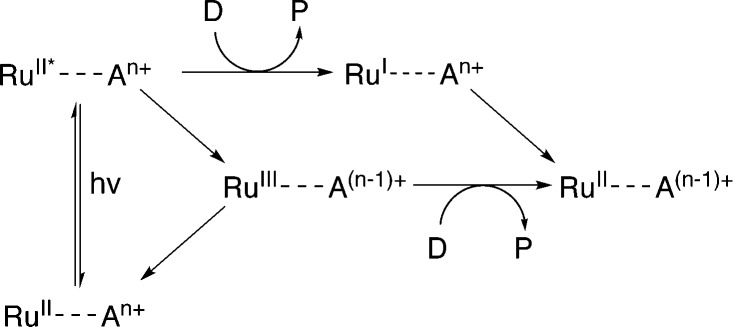
Proposed mechanisms of photosensitized reduction of an enzyme-bound cofactor upon photoexcitation of a ruthenium(ii) complex; where A = primary acceptor, D = sacrificial donor and P = oxidation products.^14^

Evidence suggests that compounds based on the related tris(2,2′-bipyrazyl)ruthenium(ii) cation, [Ru(bpz)_3_]^2+^, demonstrate much higher efficiencies in relation to their bpy analogues, primarily owing to differences in their respective electron transfer mechanism.^33,34^ Two distinct mechanisms of photosensitized reduction have been proposed, with specific pathways dependent upon the relative reduction potentials of each component (Scheme 1).^14^ Despite the increased efficiencies evident with these compounds, the bipyrazyl (bpz) systems have yet to be utilised to an appreciable extent owing in part to difficulties associated with synthesising both the pro-ligand bpz and its complexes. However, new synthetic strategies have been developed recently allowing for more extensive investigation of Ru(ii)–bpz complexes.^35^

This paper describes the use of complexes of Ru(ii) and Ir(iii) as photosensitizers for the generation of reducing equivalents in the catalytic turnover of the OYEs pentaerythritol tetranitrate reductase (PETNR)^36^ from *Enterobacter cloacae* PB2 and the thermophilic Old Yellow Enzyme (TOYE) from *Thermoanaerobacter pseudethanolicus* E39.^37^ We describe the development of a coenzyme independent, diffusion controlled multi-component system, detail the influence of individual reaction parameters upon overall efficiency, and examine the applicability of the technique towards a number of common substrates.

## 2 Experimental

### 2.1 Physical measurements

UV-vis spectra were obtained using either a Shimadzu UV-2401 PC or a Varian Cary UV-50 Bio spectrophotometer. GC analysis was performed on a Varian CP-3800 gas chromatograph equipped with a CombiPAL auto sampler and operated using the Varian Star Workstation software package. Details of the chromatography columns and running conditions are described in the ESI† (section S1.1). ^1^H NMR spectra were recorded on a Bruker UltraShield or AV-400 spectrometer. Chemical shifts are reported in parts per million relative to SiMe_4_, as referenced to the proton resonances of residual solvent using values reported in the literature.^38,39^ Elemental analyses were performed by the Microanalytical Laboratory, University of Manchester. Electrospray ionisation, MALDI and gas chromatography-mass spectrometry were performed by the University of Manchester Mass Spectrometry Service.

### 2.2 Chemical syntheses

*N*′′,*N*′′′-Dimethyl-2,2′:4,4′′:4′,4′′′-quaterpyridinium hexafluorophosphate, [Me_2_qpy^2+^](PF_6_)_2_,^40^ *trans*-Ru(bpy)(CO)_2_Cl_2_,^41^ [Ru(bpz)_2_(L–L)]Cl_2_, (where L–L = 4,4′-diamino-2,2′-bipyridine, (dNH_2_bpy), 4,4′-di-*tert*-butyl-2,2′-bipyridine, (d^*t*^Bubpy), 4,4′-dichloro-2,2′-bipyridine, (dClbpy) or bpz), [Ru(bpz)_2_(Me_2_qpy^2+^)]Cl_4_, [Ir(Me-2,2′-bpy)_2_(bpy)]Cl_3_ (Me-2,2′-bpy = (1-methyl-2-(2-pyridyl)pyridin-3-yl-ium-C^4^,*N*′) and [Ir(Me-3,2′-bpy)_2_(dCF_3_bpy)]Cl_3_ (Me-3,2′-bpy = (1-methyl-3-(2-pyridyl)pyridin-4-yl-ium-C^4^,*N*′; dCF_3_bpy = 4,4′-di-trifluoromethyl-2,2′-bipyridine) were prepared according to literature precedent.

#### [Ru(bpy)(Me_2_qpy^2+^)_2_](PF_6_)_6_

*trans*-Ru(bpy)(CO)_2_Cl_2_ (120 mg, 0.31 mmol), [Me_2_qpy^2+^](PF_6_)_2_ (441 mg, 0.70 mmol) and trimethylamine-*N*-oxide (210 mg, 2.80 mmol) were added to a round bottomed flask and the vessel purged with argon. Argon-sparged 2-methoxyethanol (10 mL) was then added *via* a syringe and the mixture refluxed for 24 h. Upon cooling to room temperature, solvents were removed by rotary evaporation and the residue redissolved in a minimum of deionized water. The resultant suspension was filtered to remove unreacted starting materials, and an excess of solid NH_4_PF_6_ added to the filtrate to precipitate the crude product. The material was isolated by vacuum filtration and purification was effected by column chromatography (SiO_2_; 0.1 M NH_4_PF_6_ in MeCN). The resultant fractions were evaporated to dryness, the product washed extensively with ice-cold deionized water and then dried *in vacuo* to give a dark red powder. Yield: 109 mg (19%). ^1^H-NMR: *δ*_H_ (400 MHz, CD_3_CN) 9.04 (2H, t, *J* = 1.9 Hz), 8.82 (2H, d, *J* = 6.6 Hz), 8.81 (2H, d, *J* = 6.6 Hz), 8.59 (1H, d, *J* = 8.4 Hz), 8.44 (2H, d, *J* = 7.2 Hz), 8.42 (2H, d, *J* = 7.2 Hz), 8.16 (1H, td, *J* = 8.0, 1.4 Hz), 8.09 (1H, d, *J* = 6.2 Hz), 8.04 (1H, d, *J* = 6.2 Hz), 7.87–7.81 (overlapping H), 7.49 (1H, ddd, *J* = 7.6, 5.8, 1.2 Hz), 4.39 (3H, s), 4.38 (3H, s). Anal. calcd (%) for C_54_H_48_F_36_N_10_P_6_Ru; C, 35.9; H, 2.7; N, 7.8. Found C, 35.7; H, 2.5; N 7.5.

#### [Ru(bpy)(Me_2_qpy^2+^)_2_]Cl_6_

To a solution of [Ru(bpy)(Me_2_qpy)_2_](PF_6_)_6_ in acetone, an excess of solid [N^*n*^Bu_4_]Cl was added and the precipitate collected by vacuum filtration. The solid was washed with acetone and diethyl ether, then dried *in vacuo* to give the chloride salt in near quantitative yield. ^1^H-NMR: *δ*_H_ (400 MHz, CD_3_OD) 9.75 (2H, dd, *J* = 4.9, 1.5 Hz), 9.13 (4H, t, *J* = 6.9 Hz), 8.85 (4H, dd, *J* = 6.9, 4.8 Hz), 8.82 (1H, d, *J* = 8.1 Hz), 8.31 (1H, d, *J* = 6.1 Hz), 8.23 (1H, td, *J* = 7.9, 1.4 Hz), 8.17–8.11 (3H), 8.02 (1H, d, *J* = 4.9 Hz), 7.59 (1H, ddd, *J* = 7.6, 5.8, 1.2 Hz), 4.51 (3H, s), 4.49 (3H, s).

### 2.3 General enzymatic procedures

The C-terminally histidine-tagged enzymes PETNR-His_8_ and TOYE-His_6_ were produced and purified as described previously.^37,42^ PETNR variants Q241C, G301C and R324C were generated by the QuikChange site directed mutagenesis protocol.^42^ Oligonucleotide sequences and PCR cycles are listed in the ESI† (S1.2), and the cloning protocols were followed as described in prior mutagenesis studies.^42^ The recombinant PETNR variants were produced and purified as described for wild-type PETNR-His_8_.^42^ Enzyme concentrations were determined using the following extinction coefficients: PETNR (*ε*_464_ = 11.3 × 10^3^ M^–1^ cm^–1^) and TOYE (*ε*_456_ = 11.3 × 10^3^ M^–1^ cm^–1^). Prior to use, all enzymes and buffer solutions were deoxygenated by passage through a BioRad 10DG column equilibrated in anaerobic reaction buffer. All reactions were set-up in an oxygen-free environment (<5 ppm of O_2_), using a Belle Technology anaerobic chamber.

### 2.4 Spectrophotometric assays

Samples were prepared anaerobically in a 10 mm quartz cuvette (1 mL) and enclosed with a suba-seal. Standard reactions (1 mL) were composed of buffer (5 mM triethanolamine (TEA)) pH 7.0 containing enzyme (10 μM), photosensitizer (20 μM) and methyl viologen dichloride ([MV^2+^]Cl_2_; 0.5 mM). The reactions were initiated by illumination with a Schott 1500 LCD lamp fitted with a 360 nm long-pass filter. The UV-vis spectra (250–800 nm) of each reaction were recorded every 1, 5 or 10 min for 1–6 h. The turnover frequency (TOF) of each reaction was calculated under non-steady state conditions.^43^

### 2.5 Aqueous light-driven reactions

Samples were prepared anaerobically in a 5 mL vial and sealed with a suba-seal and parafilm. Typical reactions (3.5 mL) were composed of buffer (5–50 mM TEA pH 6.0–10.0) containing enzyme (10 μM), substrate (5 mM in ethanol; 5% residual ethanol per reaction), photosensitizer (5–100 μM) and an electron transfer mediator (0.1 mM). The reactions were illuminated, as described above, and periodically samples (500 μL) were collected *via* syringe under a positive pressure of nitrogen. The samples were extracted with ethyl acetate (500 μL) containing limonene (0.5% v/v) as an internal standard. The organic phase was separated and dried over anhydrous MgSO_4_ prior to analysis by gas chromatography. Products were identified by comparison of retention times with authenticated standards.

### 2.6 Biphasic reactions

Standard OYE biphasic reactions were performed using an enzyme-coupled NADPH regeneration system. Reactions were carried out in 2 mL screw top vials sealed and secured with parafilm. A typical reaction aqueous phase (1 mL) was composed of buffer (50 mM, K_3_PO_4_, pH 8.0) containing enzyme (10 μM), NADP^+^ (10 μM), glucose 6-phosphate (15 mM) and glucose 6-phosphate dehydrogenase (10 units). The substrate (200 μL; 25 mM) was added directly from a stock solution in organic solvent. The reactions were illuminated, as described above, and agitated at 450 rpm under ambient temperature for 24 h. The reaction mixture was extracted with 800 μL ethyl acetate containing limonene 5% (v/v) as an internal standard and processed for GC analysis as described above. NADPH-independent, light-driven biphasic reactions were performed in a similar manner, except that the aqueous phase (1 mL) was composed of buffer (50 mM TEA pH 8.0) containing enzyme (10 μM), photosensitizer (20 μM) and [MV^2+^]Cl_2_ (0.1 mM). The remaining reaction steps were performed as for NADPH-coupled biphasic reactions.

## 3 Results and discussion

### 3.1 Spectral analysis of photoredox cycles

The photosensitizer efficiency was evaluated initially using UV-vis spectroscopy by monitoring the absorption at *ca.* 464 nm (change in the redox state of the FMN cofactor). The initial photosensitizer chosen was [Ru(bpz)_2_(dClbpy)]Cl_2_,^35^ due to its photophysical and electrochemical properties and ease of ligand and complex synthesis. Triethanolamine (TEA) was employed as the reaction buffer/sacrificial electron donor, as it is known to act efficiently with the related sensitizer [Ru(bpz)_3_]Cl_2_.^34^ All reactions were performed anaerobically to avoid quenching of the excited sensitizer and reduced transfer mediators, and to prevent unproductive OYE reduced flavin reoxidation.^42^ Unfortunately, only minor spectral changes were recorded upon illumination of a solution of [Ru(bpz)_2_(dClbpy)]Cl_2_ (20 μM) and PETNR (10 μM), the magnitude of which were insufficient to indicate reliably successful electron transfer to the cofactor. Due to spectral overlap of FMN with the metal-to-ligand charge transfer (MLCT) absorbance bands of the complex, it was unclear whether the spectral changes were due to enzyme reduction and/or photobleaching of [Ru(bpz)_2_(dClbpy)]^2+^. Therefore, the addition of the electron transfer mediator methyl viologen (as *N*,*N*′-dimethyl-4,4′-bipyridinium dichloride; [MV^2+^]Cl_2_) was added to subsequent reactions. This mediator has been shown previously to act efficiently in the catalytic turnover of multiple OYEs.^22,30^

**Fig. 1:**
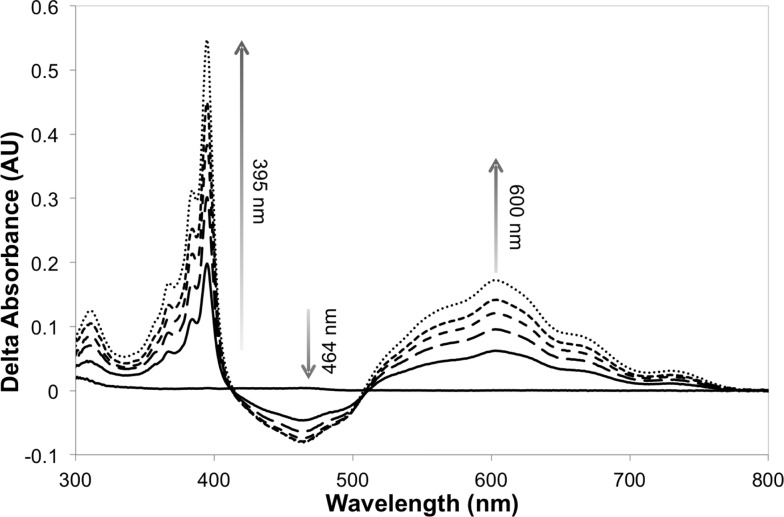
Difference spectra of the reaction between illuminated [Ru(bpz)_2_(dClbpy)]Cl_2_, [MV^2+^]Cl_2_ and PETNR, showing the accumulation of MV^+^˙ and concomitant reduction of the enzyme-bound cofactor. Samples containing [Ru(bpz)_2_(dClbpy)]Cl_2_ (20 μM), [MV^2+^]Cl_2_ (0.5 mM) and PETNR (10 μM) in TEA (5 mM, pH 7), and were illuminated with a 150 W halogen lamp fitted with a 360 nm long-pass filter. Spectra were recorded over 50 min at *ca.* 10 min intervals.

The addition of [MV^2+^]Cl_2_ (0.5 mM) to an illuminated solution of [Ru(bpz)_2_(dClbpy)]Cl_2_ (20 μM) in TEA buffer resulted in rapid electron transfer to yield the reduced cationic blue radical of methyl viologen (MV^+^˙), as indicated by an increase in absorbance at 395 nm and 600 nm (results not shown). This radical persisted for many minutes after illumination had ceased, although a slow reoxidation occurred due to trace oxygen contamination (<5 ppm). The addition of wild-type PETNR led to decreases in both the absorbance *ca.* 464 nm and the rate of MV^+^˙ accumulation (Fig. 1). The decrease in absorbance at 464 nm is characteristic of the reduction of enzyme-bound FMN to FMNH_2_, indicating successful transfer of reducing equivalents from illuminated [Ru(bpz)_2_(dClbpy)]^2+^ to PETNR *via* MV^2+^. Complete enzyme reduction was achieved after *ca.* 50 min of illumination, as indicated by no further decreases at 464 nm. Exposure of the reaction to air resulted in the immediate quenching of both MV^+^˙ and FMNH_2_ by oxygen.

### 3.2 Light-driven biotransformations

Further reactions were performed with both wild-type enzymes and three variant forms of PETNR (Q241C, G301C and R324C) in the presence of cyclohexen-2-one to see if light-driven enzymatic reduction of alkenes could be achieved. Anaerobic reactions were illuminated with a 150 W halogen lamp fitted with a 360 nm long-pass filter to minimize direct photochemical reactions of the substrate and/or mediator. Activity was monitored by the direct detection and quantification of both substrate loss (% conversion; conv.) and product cyclohexanone generation (% yield) by GC analysis (Table 1 and ESI† Fig. S1). The TOF indicates the relative rates of the reactions.

**Table 1:** Activity of wild-type and variant OYEs with cyclohexen-2-one using a light-driven hydride source

Enzyme	TOF^*a*^	Conv.^*b*^ [%]	Yield^*b*^ [%]
TOYE	121.5	100	>99
PETNR	100.4	91	89
PETNR_Q241C_	80.8	79	78
PETNR_G301C_	81.1	88	85
PETNR_R324C_	137.4	100	>99

^*a*^Determined after 120 min.

^*b*^Determined by GC analysis after 240 min.

**Scheme 2:**
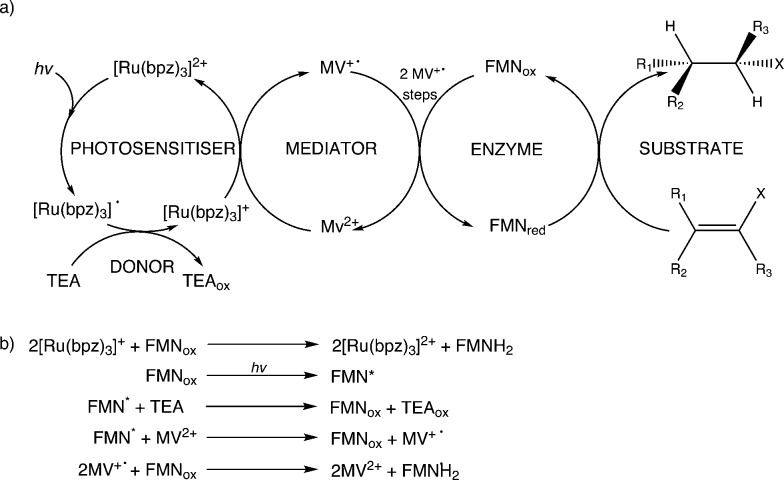
Electron transfer processes proposed to contribute towards enzyme reduction in the light-driven biocatalytic cycles of PETNR and TOYE. a) Predominant and b) minor electron transfer processes leading to substrate hydrogenation.

All reactions showed high levels of product accumulation after 4 h indicating successful coupled electron transfer between the photosensitizer and the enzymes (Scheme 2a). Complete conversion and high TOF values were detected with both TOYE and variant PETNR_R324C_. The difference in activities may be due in part to the accessibility of reduced MV^+^˙ to the FMN of the enzymes. A comparison of the X-ray crystal structures of the two enzymes shows TOYE has a larger active site than PETNR.^37,44^ This may increase the interaction between reduced MV^+^˙ and FMN, allowing more efficient electron transfer and thereby a higher turnover rate. In the case of PETNR variants, predicted surface models of PETNR_R324C_ suggest the exchange of the comparatively large arginine side chain for cysteine leads to a significant reduction in the steric bulk near the xylene ring of FMN, compared to wild-type and variants Q241C and G301C (ESI† Fig. S2). This may result in an increase in the surface exposure of the FMN to the bulk water, potentially improving the access of reduced MV^+^˙ to the FMN.

Non-illuminated reactions showed minimal to no catalytic turnover, with any product observed attributed to illumination of the sample during reaction setup, as no further accumulation of the product was noted beyond the initial reading. Minor non-enzymatic side reactions (<5% yield) were seen, possibly due to the direct reduction of the substrate by the photosensitizer or mediator. Slow direct electron transfer from the photosensitizer to the enzyme bound cofactor was seen in the absence of MV^2+^ (*ca.* 4% and 8% yields for PETNR_R324C_ and TOYE, respectively). Minor activity was detected with TOYE without an exogenous photosensitizer, suggesting a photoreduction of MV^2+^ in the presence of free flavin.^45^ The exclusivity of such turnover to assays of TOYE is primarily attributed to the increased accessibility of the bound cofactor to both MV^2+^ and the sacrificial donor.

In summary, the main electron transfer pathway proceeds *via* photoexcitation of the photosensitizer followed by successive reductive and oxidative steps between TEA, methyl viologen and enzyme bound flavin (FMN) to the alkene substrate (Scheme 2a). Additional proposed minor electron transfer steps include a direct electron transfer from the reduced photosensitizer to FMN, and photoexcitation of the enzyme bound FMN and subsequent reductive quenching of the excited triplet state by TEA (Scheme 2b).

### 3.3 Photosensitizer screening

**Scheme 3:**
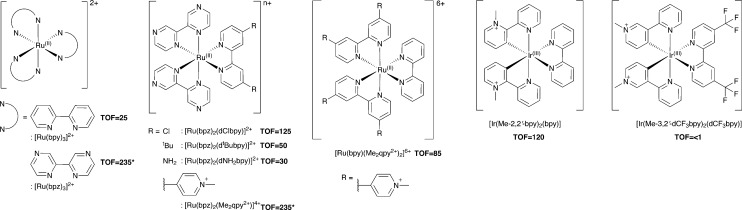
Photosensitizers used in this study. TOFs of TOYE with each photosensitizer after 120 min (60 min for *) are shown in bold.

Additional Ru(ii) complexes were tested as photosensitizers with both PETNR_R324C_ and TOYE; [Ru(bpz)_2_(L–L)]^2+^ (where L–L = dNH_2_bpy, d^t^Bubpy, dClbpy or bpz), [Ru(bpz)_2_(Me_2_qpy^2+^)]^4+^ and [Ru(bpy)(Me_2_qpy^2+^)_2_]^6+^ (Scheme 3). Also, [Ir(Me-2,2′-bpy)_2_(bpy)]^3+^ and [Ir(Me-3,2′-bpy)_2_(dCF_3_bpy)]^3+^ were chosen from a series of highly water soluble Ir(iii) cyclometalated complexes (Scheme 3).^46,47^ [Ru(bpy)_3_]^2+^ was employed as a control standard, and Cl^–^ salts were used in all cases. In each case, reactions were performed with cyclohexen-2-one as the enzyme oxidative substrate. Reactions were analysed by GC to determine the TOF (Scheme 3 for TOYE), conversion and product yields (ESI† Table S1 and Fig. S3).

Comparative reactions with Ru(ii) sensitizers show that the TOF correlates with the potential of the Ru^(III/II)^ couple (ESI† Fig. S3). This trend is illustrated by the dramatic increase in TOF with TOYE on moving from [Ru(bpy)_3_]^2+^ to [Ru(bpz)_3_]^2+^ or [Ru(bpz)_2_(Me_2_qpy^2+^)]^4+^ (Scheme 3). Reactions of both OYEs with photosensitizers [Ru(bpz)_2_(dClbpy)]^2+^, [Ru(bpz)_2_(Me_2_qpy^2+^)_2_]^4+^, show >99% product yields, while others are less successful (ESI† Table S1). The relatively enhanced activities of the Me_2_qpy^2+^ complexes may be attributed in part to more efficient light absorption, because their MLCT bands are significantly more intense than those of the other complexes.

It is assumed for [Ru(bpz)_3_]^2+^ and related cations that electron transfer proceeds *via* initial reduction of the excited sensitizer. Therefore, yields should increase on enhancing the oxidising ability of the excited complex as reductive quenching by TEA becomes more favourable. In contrast, the electron transfer processes of [Ru(bpy)_3_]^2+^ likely proceed *via* an initial oxidative quenching of the excited state by MV^2+^ due to the relative differences in redox potentials. The previously reported quantum yields of MV^+^˙ formation are relatively low in comparison to the bpz analogues, and are attributed to rapid back electron transfer to the oxidized complex.^34^ Considering that [Ru(bpz)_2_(dNH_2_bpy)]^2+^ exhibits similar *E*(Ru^III/II^), +1.43 V (ref. 40) compared to +1.32 V *vs.* Ag–AgCl for [Ru(bpy)_3_]^2+^, an equivalent mechanism could explain the relative ineffectiveness of this sensitizer. Considering the complete lack of activity in the systems of both of these sensitizers with PETNR, the observed TOFs with TOYE may result from the alternate electron transfer pathways described in Scheme 2b.

Definitive mechanisms of electron transfer using cyclometalated Ir(iii) photosensitizers have yet to be determined.^48^ However, consideration of the redox potentials of the highly effective [Ir(Me-2,2′-bpy)_2_(bpy)]^3+^ suggests initial reductive quenching of the excited sensitizer. A similar mechanism might be expected for [Ir(Me-3,2′-bpy)_2_(dCF_3_bpy)]^3+^, but its inactivity suggests otherwise. At present it is unknown whether the lack of turnover may result from the poor absorbance of this sensitizer at *λ* ≥ 360 nm or from a potential instability of the complex in the excited state.

In some cases a mild level of product decomposition was detected during extended periods of illumination after complete consumption of the substrate. For example, in the assays with [Ru(bpz)_3_]^2+^, reductions of *ca.* 5–8% in yield were observed over 2 h after complete conversion had been achieved. This may be due to the relatively high oxidising ability of the complexes, or exposure of the products to high concentrations of MV^+^˙ for an extended period of time leading to unwanted side reactions.

### 3.4 Reaction optimisation

Given the successful photosensitizer-driven bioreduction of cyclohexen-2-one by both TOYE and PETNR, reaction optimisation studies were performed to enhance TOFs and ultimately product yields. Reaction parameters modified are the photosensitizer concentration, sacrificial electron donor concentration, pH dependence and the irradiation wavelength. Full discussion and data describing the optimisation studies can be found in the ESI,† and only a summary is given here.

For both PETNR and TOYE, product yield increases with increasing [Ru(bpz)_2_dClbpy]Cl_2_ concentration, in the presence of an excess of TEA and MV^2+^, with an optimal concentration of 20 μM (ESI† Fig. S5a; Table S2 and Fig. S4). Concentration dependence studies with TEA show maximal yields at 25 mM TEA (ESI† Table S3, Fig. S6 and S5b). These results are consistent with the proposed mechanism where turnover is limited by the rate of generation of the reduced sensitizer upon quenching of the excited state by TEA (Scheme 2a). This is in contrast to direct light-driven flavin reduction mechanisms of phenylacetone monooxygenase from *Thermobifida fusca* and the OYE YqjM from *B. subtilis*. Both enzymes exhibited initial rates independent of the donor concentration.^15,20^

The pH optimisation studies of PETNR and TOYE with cyclohexen-2-one and photosensitizers [Ru(bpz)_2_(dClbpy)]Cl_2_ and Ir(Me-2,2′-bpy)_2_(bpy)]Cl_3_ show maximal yields in the pH range of 8–10 (ESI† Table S4, Fig. S5c and S7). The [Ru(bpz)_2_(L–L)]^2+^ complexes show a greater pH dependence than the Ir(iii) compounds, with a dramatic decrease in activity with increasing acidity. This observation can be ascribed to deactivation of the photosensitizer upon protonation of the bpz ligands.^45^

The varying absorption profiles and redox properties of transition metal complexes suggest that different sensitizers may be selectively excited at specific wavelengths, enabling a finer level of control over light-triggered reactions. Experiments along these lines used long pass optical filters (530, 460, 360 and 305 nm), which attenuate light of higher energy and allow for selective excitation of transitions occurring at longer wavelengths (ESI† Fig. S8, Table S5 and Fig. S9). On illumination at *λ* ≥ 460 nm with PETNR_R324C_, [Ru(bpz)_2_(dClbpy)]Cl_2_ shows very high activity, substantially larger than that of [Ru(bpy)(Me_2_qpy^2+^)_2_]Cl_6_ (ESI† Fig. S5d), despite the more intense MLCT absorption of the latter. Under the same conditions, the weakly absorbing [Ir(Me-2,2′-bpy)_2_(bpy)]Cl_3_ shows some activity, while [Ir(Me-3,2′-bpy)_2_(dCF_3_bpy)]Cl_3_ is completely inactive due to a lack of any absorption above 460 nm.

### 3.5 Substrate specificity

To probe the scope of the system, reactions with four distinct substrate classes were performed (α,β-unsaturated aldehyde/ketones, maleimides, nitroalkenes and terpenoids) using TOYE or PETNR_R324C_ and [Ru(bpz)_2_(dClbpy)]Cl_2_ as photosensitizer (Table 2). Under these conditions, MV^+^˙ accumulation depends on the enzyme activity towards the substrate, so in most cases the rate-limiting step is enzyme-dependent rather than external hydride supply.

**Table 2:** Reduction of activated alkenes by TOYE and PETNR_R324C_ in light-driven biocatalytic systems employing [Ru(bpz)_2_(dClbpy)]Cl_2_ as the photosensitizer

Substrate	Enzyme	TOF^*a*^	Conv.^*b*^ [%]	Yield^*b*^ [%]	ee^*b*^ [%]
Cinnamaldehyde	TOYE	110	93	80	—
	PETNR_R324C_	130	100	85	—
α-Methylcinnamaldehyde	TOYE	190	100	68	9 (*S*)
	PETNR_R324C_	36	56	22	37 (*S*)
2-Methylpentenal	TOYE	130	100	73	6 (*S*)
	PETNR_R324C_	105	100	65	85 (*S*)
Ketoisophorone	TOYE	260*	100	88	35 (*R*)
	PETNR_R324C_	190	100	95	40 (*R*)
*N*-Phenyl-2-methylmaleimide	TOYE	125	100	82	>99 (*R*)
	PETNR_R324C_	10	81	10	>99 (*R*)
*trans*-β-Nitrostyrene	TOYE	—	100	0	—
	PETNR_R324C_	—	100	0	—
(*S*)-Carvone	TOYE	18	27	6	98 (2*R*,5*S*)
	PETNR_R324C_	15	24	5	>99 (2*R*,5*S*)

^*a*^Turnover frequency determined after 120 min.

^*b*^Determined by GC analysis after a 240 min reaction, except reactions indicated by * were analysed after 60 minutes. Conv. = conversion.

The most productive reactions (80–95% yield) are with the classic OYE substrates cinnamaldehyde and ketoisophorone (Table 2).^2^ In the latter case, the product ee's are modest (35 or 40% (*R*)), partially due to water-mediated product racemisation.^37^ Also, ketoisophorone undergoes a non-enzymatic reduction upon direct electron transfer from MV^+^˙ or the reduced photosensitizer to generate racemic levodione in *ca.* 80% yields. TOYE shows higher reactivity than PETNR_R324C_ (68% *vs.* 22% yield, respectively).

PETNR is susceptible to substrate inhibition with α-methylcinnamaldehyde in the low millimolar range,^49^ and the poor ee's with this substrate (9 or 37%; Table 2) are attributed to non-enzymatic water-mediated product racemisation, typical of compounds with a stereogenic centre at Cα.^49^ This loss of stereochemical information most probably occurs *via* tautomerisation; more specifically, a base-catalysed enolization that is promoted upon deprotonation of Cα.

Another good OYE substrate, *N*-phenyl-2-methylmaleimide, is reduced with high enantiomeric purity (>99% (*R*); Table 2). However, PETNR_R324C_ generates poor yields (10%), with high substrate and/or succinimide product degradation when compared to the TOYE reaction. This inefficiency is due to an unidentified non-enzymatic side reaction, which accounts for *ca.* 50% of the substrate consumption in control assays performed in the absence of enzyme.

These observations are consistent with the relative rates of catalytic turnover with the two enzymes using NAD(P)H as the hydride source.^37,49^ In contrast, there is a large disparity in ee between TOYE and PETNR_R324C_-catalysed reductions of 2-methylpentenal (6 *vs.* 85% (*S*), respectively; Table 2). Given the similar conversions and yields, this enantiopurity difference may be due to variations in substrate orientation within the active sites.

Reactions with the monoterpenoid (*S*)-carvone generate poor yields, due to loss of activity after the first hour (Table 2). Similar behaviour is observed with the related substrate perillaldehyde, but with considerably lower overall product formation and activity ceasing within 30–40 min. This inhibition may be due to an accumulation of dihydrocarvone or possibly a by-product. In the case of *trans*-β-nitrostyrene, complete substrate consumption is achieved with no product formation detectable by GC analysis (Table 2). Decomposition probably occurs *via* direct reduction of the nitro group by MV^+^˙, as previously described for biphasic reactions of nitro aromatics in the presence of dioctylviologen, with sodium dithionite as the primary electron donor.^50^

### 3.6 Biphasic assays

**Fig. 2:**
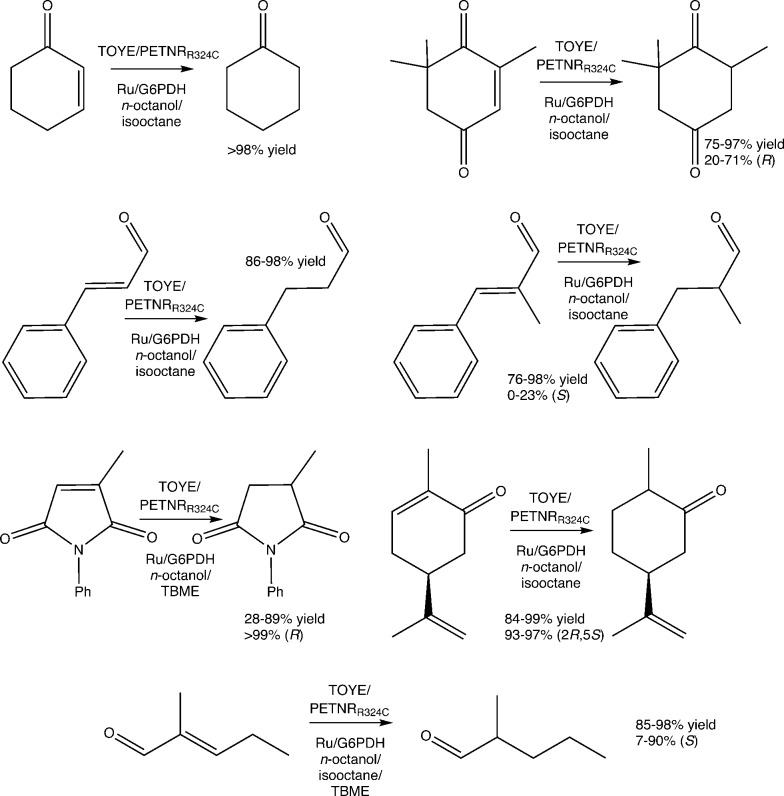
Biphasic reduction of α,β-unsaturated alkenes by TOYE or PETNR_R324C_ using either a photosensitizer or a NADP^+^/G6PDH cofactor recycling system as the hydride supply. Conditions for assays containing a photosensitizer: enzyme (10 μM), [Ru(bpz)_2_(dClbpy)]Cl_2_ ({Ru} 20 μM) and [MV^2+^]Cl_2_ (0.1 mM) in TEA buffer (1.0 mL, 50 mM, pH 8.0). Conditions for assays containing G6PDH regeneration system: enzyme (10 μM), NADP^+^ (10 μM), glucose-6-phosphate (15 mM) and G6PDH (10 U) in phosphate buffer (1.0 mL, 50 mM, pH 8.0). Substrate added as a solution in the indicated solvent (25 mM, 200 μL). Assays were undertaken at RT for 24 h at 450 rpm.

Reactions were performed with TOYE or PETNR_R324C_ under biphasic conditions to minimise water-mediated side reactions and improve substrate/product solubility (ESI† Table S6). The solvents iso-octane, *n*-octanol and *tert*-butylmethyl ether (TBME) have been used previously in biphasic reactions with of a variety of OYEs.^30,36,51^ Experiments over 24 h involved seven oxidative substrates, with either a photosensitizer ([Ru(bpz)_2_(dClbpy)]Cl_2_; 50 mM TEA) or a NADP^+^/glucose-6-phosphate dehydrogenase (G6PDH) cofactor recycling system (Fig. 2).

In almost all cases, reactions involving PETNR_R324C_ with NADP^+^/G6PDH and cosolvent TBME generate considerably lower product yields (*e.g.* 14% *vs.* >98% yield of cyclohexanone in other co-solvents; ESI† Table S6), due to a significant increase in the precipitation of protein (PETNR_R324C_ and/or G6PDH). The improved performance of TOYE with G6PDH in TBME (Table S6†) is likely due to its enhanced solubility over PETNR and G6PDH.^37^

Apart from reaction in TBME, product yields from the biphasic reduction of cyclohexen-2-one, ketoisophorone, (*S*)-carvone or cinnamaldehyde are often remarkably similar despite changing co-solvent, hydride source and OYE type (ESI† Table S6). For example, (*S*)-carvone gives near quantitative yields of dihydrocarvone with excellent diastereomeric excess in most cases. These results are due to avoidance of the inhibitory effects observed in the homogenous reactions, because the organic phase acts as both a substrate reservoir and product sink. Also the biphasic reactions of cinnamaldehyde, α-methyl-cinnamaldehyde and 2-methylpentenal all display increased yields compared to the homogenous reactions. Unfortunately, biphasic reactions with *N*-phenylmethylmaleimide in isooctane are precluded by poor substrate solubility.

The addition of co-solvents in some cases moderately improves the product enantiopurity. For example, the combinations of TOYE/G6PDH/TBME and PETNR_R324C_/G6PDH/TBME yield (*R*)-levodione from ketoisophorone with 71–73% ee. Other combinations give poor product enantiopurity, such as TOYE with the photosensitizer in *n*-octanol (20% ee). The highest variability in product yields occurs with *N*-phenyl methylmaleimide, varying from 28–89% yields, but with exceptionally high product ees (>99%) in all cases. Biphasic reactions with α-methylcinnamaldehyde do not show any improvement in ee (0–23%) from values reported for PETNR previously.^49^ In contrast, near quantitative yields are obtained with most combinations of enzyme/hydride source/solvent with 2-methylpentenal (ESI† Table S6), although ees varied considerably (7–90%). Another general observation is the maintenance of enzyme-mediated enantioselectivities seen in the homogeneous reactions, such as the distinct decrease in ee (7–30%) of the product from the reaction of 2-methylpentenal with TOYE compared to PETNR (75–90%).

## 4 Conclusions

This study highlights the potential usefulness of photosensitive transition metal complexes as electron donors in OYE-catalysed α,β-unsaturated alkene reduction, when coupled with sacrificial electron donors and electron transfer mediators. Both PETNR and TOYE reduce a broad range of substrates under either aqueous or biphasic conditions. The product yields and enantiopurity are often at least comparable to reactions using NADPH as the hydride donor. Further reaction optimisation studies are likely to improve yields and/or product enantiopurity even further, such as varying the photosensitizer type, pH and relative reaction component concentrations.

Given the potential commercial applicability of this cofactor regeneration system, further developments such as fine-tuning the photosensitizer excitation wavelength could lead to the development of a photosensitized cascading reaction. This could lead to the development of multiple sequential chemical transformations, with selective activation of specific steps. Therefore, there is great potential in the development of practical light-driven biocatalytic systems using transition metal complexes, providing an alternative to the use of costly redox coenzymes or reliance upon the enzyme-based cofactor regeneration systems.

## Abbreviations

bpy: 2,2′-Bipyridine
bpz: 2,2′-Bipyrazine
dMebpy: 4,4′-Dimethyl-2,2′-bipyridine
d^*t*^Bubpy: 4,4′-Di-*tert*-butyl-2,2′-bipyridine
dNH_2_bpy: 4,4′-Diamino-2,2′-bipyridine
Me_2_qpy^2+^: *N*′′,*N′′′*-Dimethyl-2,2′:4,4′′:4′,4*′′′*-quaterpyridinium
Me-2,2′-bpy: (1-Methyl-2-(2-pyridyl)pyridin-3-yl-ium-C^4^,*N*′)
Me-3,2′-bpy: (1-Methyl-3-(2-pyridyl)pyridin-4-yl-ium-C^4^,*N*′)
