# Supplementary material for: Light-driven biocatalytic reduction of α,β-unsaturated compounds by ene reductases employing transition metal complexes as photosensitizers
Source: Catal Sci Technol. 2015 Oct 26;6(1):169–77. doi: 10.1039/c5cy01642h (PMC4786955; doi:10.1039/c5cy01642h)
Supplement: Supplementary file 1 [file CY-006-C5CY01642H-s001.pdf]

Electronic Supplementary Material (ESI) for Catalysis Science & Technology.
This journal is © The Royal Society of Chemistry 2015

SUPPORTING INFORMATION

Light-Driven Biocatalytic Reduction of α,β-Unsaturated Compounds by Ene Reductases Employing Transition Metal Complexes as Photosensitizers

Martyn K. Peers,[a] Helen S. Toogood,[a] Derren J. Heyes,[a] David Mansell,[a] Benjamin J. Coe[b] and Nigel S. Scrutton [a]†

[a] *Faculty of Life Sciences, University of Manchester, 131 Princess Street, Manchester, M1 7DN (UK)*

[b] *School of Chemistry, University of Manchester, Oxford Road, Manchester, M13 9PL (UK)*

† Corresponding author: email nigel.scrutton@manchester.ac.uk

S1 Experimental

S1.1 Analytical procedures

S1.1.1 Determination of percentage yields and conversions

S1.1.2 Determination of enantiomeric excess

S1.2 Oligonucleotides and PCR reaction conditions

S2 Results and discussion

S2.1 General data

Fig. S1 Light-driven bioreduction of 2-cyclohexenone by a variety of OYEs using $[Ru(bpz)_2(dClbpy)]Cl_2$ as a photosensitizer.

Fig. S2 Predicted surface model of the active site of PETNR (top) and variant $PETNR_{R324C}$ (bottom).

Table S1 Influence of modifications made to the photosensitizer upon the activity of light-driven biocatalytic systems in the reduction of 2-cyclohexenone by PETNR or TOYE.

Fig. S3 Influence of the photosensitizer upon the light-driven bioreduction of cyclohexen-2-one by TOYE (top). Plot of potential of the Ru(III/II) couple (vs Ag–AgCl) vs TOF obtained in the assays of both TOYE and $PETNR_{R324C}$ (bottom).

S2.2 Effect of the photosensitizer concentration on OYE activity

Table S2 Impact of changing photosensitizer concentration upon the rates of light-driven biocatalytic reduction of cyclohexen-2-one by PETNR or TOYE.

Fig. S4 Influence of photosensitizer concentration upon the light-driven bioreduction of cyclohexen-2-one by $PETNR_{R324C}$ (top) or TOYE (bottom).

Fig. S5 Summary of the optimisation studies of the photosensitizer-driven OYE-catalysed reduction of cyclohexen-2-one to cyclohexanone.

S2.3 Effect of the sacrificial electron donor concentration on OYE activity

Table S3 Influence of changes in sacrificial electron donor concentrations upon the rates of light-driven biocatalytic reduction of cyclohexen-2-one by PETNR or TOYE.

Fig. S6 Influence of TEA concentration upon the light-driven bioreduction of cyclohexen-2-one by $PETNR_{R324C}$ using $[Ru(bpz)_2(dClbpy)]Cl_2$ (top) or $[Ir(1\text{-}Me\text{-}2,2'\text{-}bpy^+)_2(bpy)]Cl_3$ (bottom) as the photosensitizer.

S2.4 The pH dependence of OYE activity

Table S4 The pH dependene of the light-driven biocatalytic reduction of cyclohexen-2-one by PETNR or TOYE.

Fig. S7 Influence of pH on the light-driven $PETNR_{R324C}$-catalysed reduction of cyclohexen-2-one using $[Ru(bpz)_2(dClbpy)]Cl_2$ (top) or $[Ir(1\text{-}Me\text{-}2,2'\text{-}bpy)_2(bpy)]Cl_3$ (bottom) as photosensitizer.

S2.5 Effect of the irradiation wavelength on OYE activity

Fig. S8 UV-Vis absorption spectra of $[Ru(bpy)(Me_2qpy^{2+})_2]Cl_6$, $[Ru(bpz)_2(dClbpy)]Cl_2$, $[Ir(Me\text{-}2,2'\text{-}bpy)_2(bpy)]Cl_3$ and $[Ir(Me\text{-}3,2'\text{-}bpy)_2(dCF_3bpy)]Cl_3$ recorded in deionized water.

Table S5 Influence of the wavelength of incident irradiation in the light-driven bioreduction of cyclohexen-2-one by $PETNR_{R324C}$.

Fig. S9 Influence of the photosensitizer and the wavelength of incident irradiation upon the light-driven bioreduction of cyclohexen-2-one by $PETNR_{R324C}$.

S2.6 Biotransformation data

Table S6 Biphasic reduction of various activated alkenes by PETNR or TOYE using a photosensitizer or a $NADP^+$/G6PDH co-factor regeneration system

S1 EXPERIMENTAL

S1.1 Analytical procedures

S1.1.1 Determination of percentage Yields and Conversions: GC analysis of reaction progress was performed using a DB-Wax column (30 m, 0.32 mm, 0.25 μm) and the peak areas were compared to known standards and associated calibration curves. **Cyclohexen-2-one:** split 20, flow 1.0 mL min^{-1}, injector: 220°C, detector: 250°C, temperature programme: 40°C hold for 10 min, to 210°C at 20°C min^{-1}, hold for 1 min; retention times: Substrate: 15.19 min, Product 13.66 min. **Cinnamaldehyde:** split 20, flow 2.0 mL min^{-1}, injector: 250°C, detector: 250°C, temperature programme: 100°C hold for 2 min, to 220°C at 20°C min^{-1}, hold for 5 min; retention times: Substrate: 6.55 min, Product 5.12 min. **α-Methylcinnamaldehyde:** As for cinnamaldehyde; retention times: Substrate: 6.35 min, Product: 5.04 min. **2-Methylpentenal:** split 20, flow 1.0 mL min^{-1}, injector: 220°C, detector: 250°C, temperature programme: 40°C hold for 10 min, to 200°C at 20°C min^{-1}, hold for 1 min; retention times: Substrate: 11.39 min, Product 5.61 min. **Ketoisophorone:** split 20, flow 1.0 mL min^{-1}, injector: 220°C, detector: 150°C, temperature programme: 110°C hold for 5 min, to 210°C at 20°C min^{-1}, hold for 1 min; retention times: Substrate: 7.61 min, Product 8.32 min ***N*-Phenyl-2-methylmaleimide:** split 20, flow 1.0 mL min^{-1}, injector: 220°C, detector: 250°C, temperature programme: 110°C hold for 2 min, to 240°C at 20°C min^{-1}, hold for 10 min; retention times: Substrate: 9.72 min, Product 10.95 min. **(*S*)-Carvone:** split 20, flow 1.0 mL min^{-1}, injector: 220°C, detector: 250°C, temperature programme: 60°C hold for 2 min, to 210°C at 15°C min^{-1}, hold for 3 min; retention times: Substrate: 10.83, Products: (2*R*,5*R*)-dihydrocarvone and (2*S*,5*R*)-dihydrocarvone at 10.36 and 10.56 min, respectively.

S1.1.2 Determination of Enantiomeric Excess: Absolute configurations were assigned on comparison to authentic samples of enantiomerically pure material. **2-Methylpentenal:** Rt-BDEXsa column (30 m, 0.25 mm, 0.25 μm): split 100, flow 1.0 mL min^{-1}, injector: 180°C, detector: 250°C, temperature programme: 80°C hold for 10 min, to 120°C at 4°C min^{-1}, hold for 2 min, to 180°C at 20°C min^{-1}, hold for 1 min; retention times: (*R*)-2-methylpentanal and (*S*)-2-methylpentanal at 12.19 and 13.47 min, respectively. **α-Methylcinnamaldehyde:** Chirasil-DEX CB column (25 m, 0.32 mm, 0.25 μm): split 100, flow 3.0 mL min^{-1}, injector: 250°C, detector: 150°C, temperature programme: 90°C hold for 30 min, to 180°C at 20°C min^{-1}, hold for 1 min; retention times: (*S*)-dihydrocinnamaldehyde and (*R*)-dihydrocinnamaldehyde at 27.14 and 27.64 min, respectively. **Ketoisophorone:** Chirasil-DEX CB column (25 m, 0.32 mm, 0.25 μm): split 100, flow 2.5 mL min^{-1}, injector: 250°C, detector: 250°C, temperature programme: 115°C hold for 10 min, to 180°C at 20°C min^{-1}, hold for 1 min; retention times: (*R*)-levodione and (*S*)-levodione at 7.80 and 8.25 min, respectively. ***N*-Phenyl-2-methylmaleimide:** Rt-BDEXsm column (30 m, 0.25 mm, 0.25 μm): split 100, flow 2.5 mL min^{-1}, injector: 180°C, detector: 250°C, temperature programme: 80°C hold for 1 min, to 200°C at 2°C min^{-1}, hold for 1 min; retention times: (*S*)-*N*-phenyl-2-methylsuccinimideand (*R*)-*N*-phenyl-2-methylsuccinimide at 50.65 and 50.91 min, respectively.

S1.2 Oligonucleotides and PCR reaction conditions

The following pairs of oligonucleotide sequences were used to generate PETNR variants. Nucleotides highlighted in red are mutations of the original sequence, and the triplet codon in bold indicates the subsequent amino acid modified.

PETNR$_{Q241C}$: PQ241CF – TCCCCGATCGGTACTTTC**TGT**AACGTCGACAACGGTCC; PQ241CR – GGACCG TTGTCGACGTT**ACA**GAAAGTACCGATCGGGGA

PETNR$_{G301C}$: PG301CF – GTGATTATCGGGGCG**TGT**GCGTATACGGCAGAG; PG301CR – CTCTGCCGTAT ACGC**ACA**CGCCCCGATAATCAC

PETNR$_{R324C}$: PR324CF – GCCGTGGCCTTTGGC**TGT**GACTACATTGCTAAC; PR324CR – GTTAGCAATGT AGTC**ACA**GCCAAAGGCCACGGC

The PCR reaction cycling conditions for PETNR variant generation are as follows:

1. **Initial denaturation:** 94 °C for 120 s
2. **Denaturation:** 94 °C for 15 s
3. **Annealing:** 54, 54 and 55 °C for 30 s (Q241C, G301C and R324C, respectively)
4. **Extension:** 68 °C for 270 s
5. Repeat steps 2-4 18 times
6. **Final denaturation:** 68 °C for 300 s

S2 RESULTS AND DISCUSSION

S1.1 General data

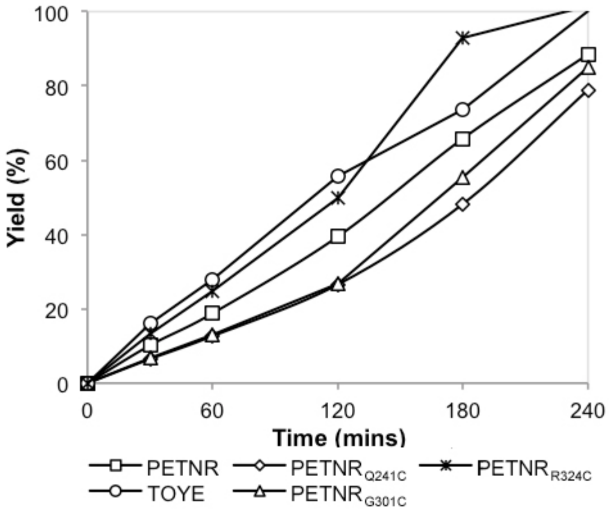

Fig. S1 Light-driven bioreduction of cyclohexen-2-one by a variety of OYEs using $[Ru(bpz)_2(dClbpy)]Cl_2$ as a photosensitizer.

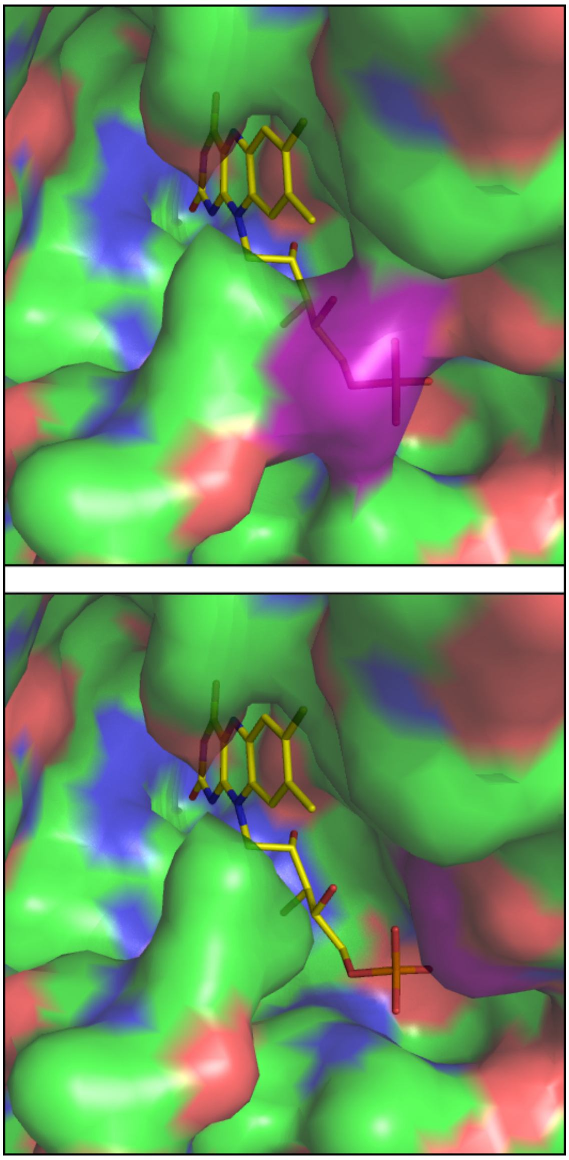

Fig. S2 Predicted surface model of the active site of PETNR (top) and variant $PETNR_{R324C}$ (bottom); R324 residue highlighted in purple. Images and predicted structural mutations generated using MacPymol.[1]

Table S1. Influence of modifications made to the photosensitizer upon the activity of light-driven biocatalytic systems in the reduction of cyclohexen-2-one by PETNR or TOYE.

Photosensitizer	Enzyme	TOF[a]	Conv.[b] [%]	Yield[b] [%]
$[Ru(bpy)_3]Cl_2$	TOYE	25	23	16
	$PETNR_{R324C}$	-	5	0
$[Ru(bpz)_3]Cl_2$	TOYE	235*	100	93
	$PETNR_{R324C}$	255*	100	95
$[Ru(bpz)_2(dNH_2bpy)]Cl_2$	TOYE	30	24	16
	$PETNR_{R324C}$	-	3	0
$[Ru(bpz)_2(d^tBubpy)]Cl_2$	TOYE	50	47	42
	$PETNR_{R324C}$	45	66	64
$[Ru(bpz)_2(dClbpy)]Cl_2$	TOYE	125	100	>99
	$PETNR_{R324C}$	135	100	>99
$[Ru(bpz)_2(Me_2qpy^{2+})]Cl_4$	TOYE	235*	100	>99
	$PETNR_{R324C}$	275*	100	>99
$[Ru(bpy)(Me_2qpy^{2+})_2]Cl_6$	TOYE	85	66	64
	$PETNR_{R324C}$	95	76	69
$[Ir(Me\text{-}2,2'\text{-}bpy)_2(bpy)]Cl_3$	TOYE	120	100	>99
	$PETNR_{R324C}$	100	100	97
$[Ir(Me\text{-}3,2'\text{-}bpy)_2(dCF_3bpy)]Cl_3$	TOYE	-	5	0
	$PETNR_{R324C}$	-	4	0

[a]Determined after 120 min. [b]Determined by GC analysis after 240 min, except reactions indicated by * which were analysed after 60 min.

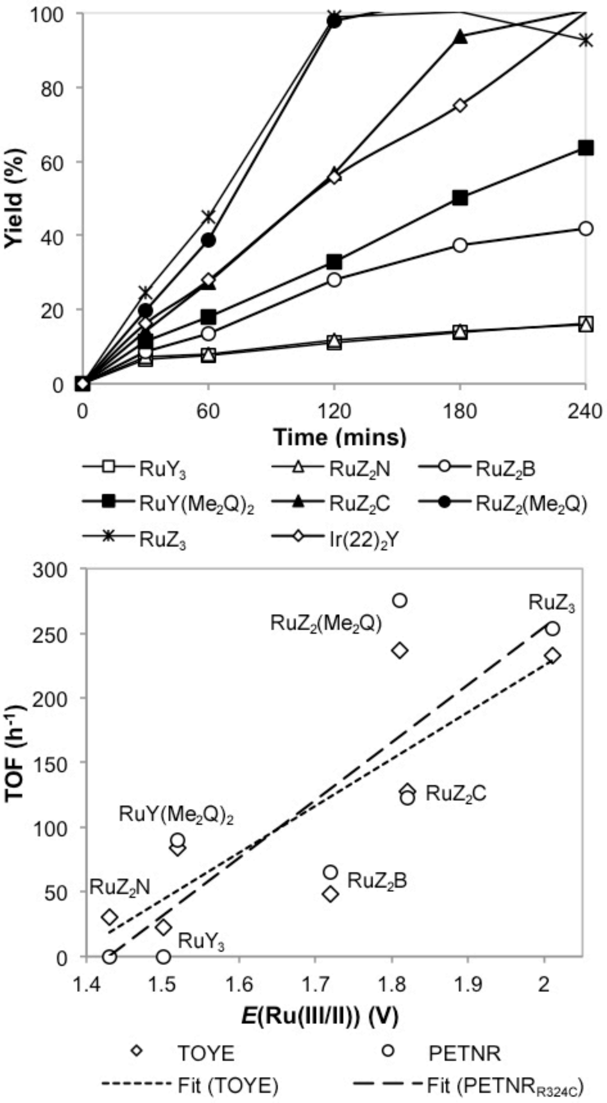

Fig. S3 Influence of the photosensitizer upon the light-driven bioreduction of cyclohexen-2-one by TOYE (top). Plot of redox potentials of the Ru(III/II) couple (vs Ag–AgCl) vs TOF obtained in the assays of both TOYE and $PETNR_{R324C}$ (bottom) (Y = bpy; Z = bpz; B = $d^{t}Bubpy$; C = dClbpy; N = dNH_2bpy; Me_2Q = Me_2qpy^{2+}).

S2.2 Effect of the photosensitizer concentration on OYE activity

Given the successful photosensitizer-driven bioreduction of cyclohexen-2-one by both TOYE and PETNR, reaction optimisation studies were performed to enhance TOF and ultimately product yields. Using $[Ru(bpz)_2(dClbpy)]Cl_2$, reactions were performed to determine the optimal levels of photosensitizer in the presence of an excess of TEA and $[MV^{2+}]Cl_2$ (Table S2 and Figure S4). For both OYEs, product yield increased with photosensitizer concentration, with an optimal concentration of 20 μM (Figure S5a).

Table S2 Impact of changing photosensitizer concentration upon the rates of light-driven biocatalytic reduction of cyclohexen-2-one by PETNR or TOYE.

Enzyme	[PS] (μM)	TOF[a]	Conv.[b] [%]	Yield[b] [%]
TOYE	5	85	86	85
	20	130	100	>99
	50	340	100	97
	100	360	100	>99
PETNR	5	45	39	37
	20	125	100	>99
	35	170	100	>99
	50	230	100	97

[a]Determined after 120 min. [b]Determined by GC analysis after 240 min.

Interestingly, while maximal cyclohexanone production was achieved with only 20 mM photosensitizer, the TOF continues to increase at higher levels. This suggests the rate of $MV^{+\bullet}$ formation is sufficient with 20 μM photosensitizer to achieve maximal product generation within 120 min, but the overall catalytic turnover is limited by non-saturating $MV^{+\bullet}$ formation. At higher photosensitizer concentrations, the rate of $MV^{+\bullet}$ formation may also show a greater dependence on other factors, such as the concentration of MV^{2+}/TEA and the binding of $MV^{+\bullet}$.

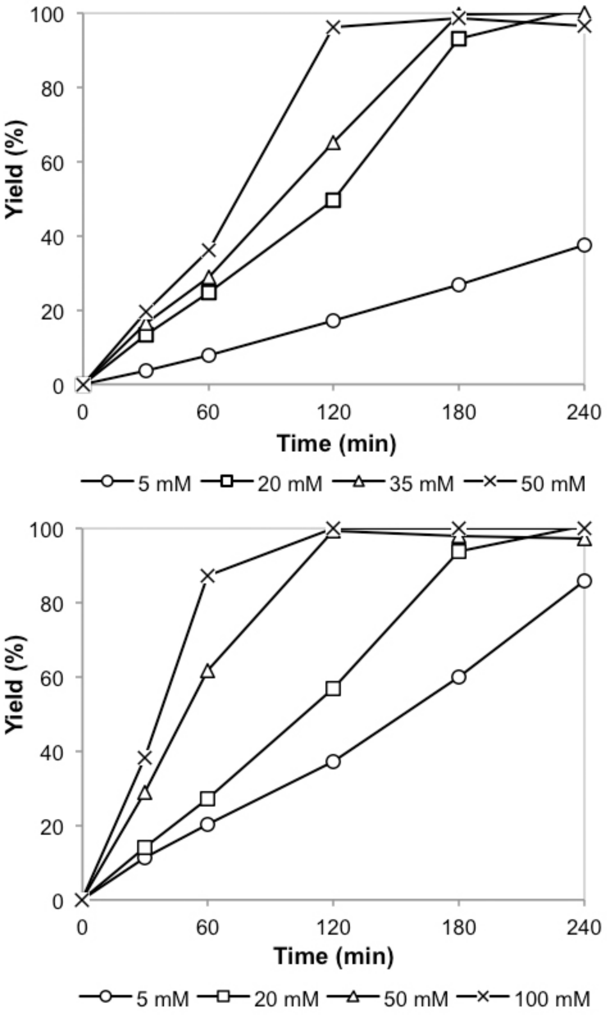

Fig. S4 Influence of photosensitizer concentration upon the light-driven bioreduction of cyclohexen-2-one by $PETNR_{R324C}$ (top) or TOYE (bottom).

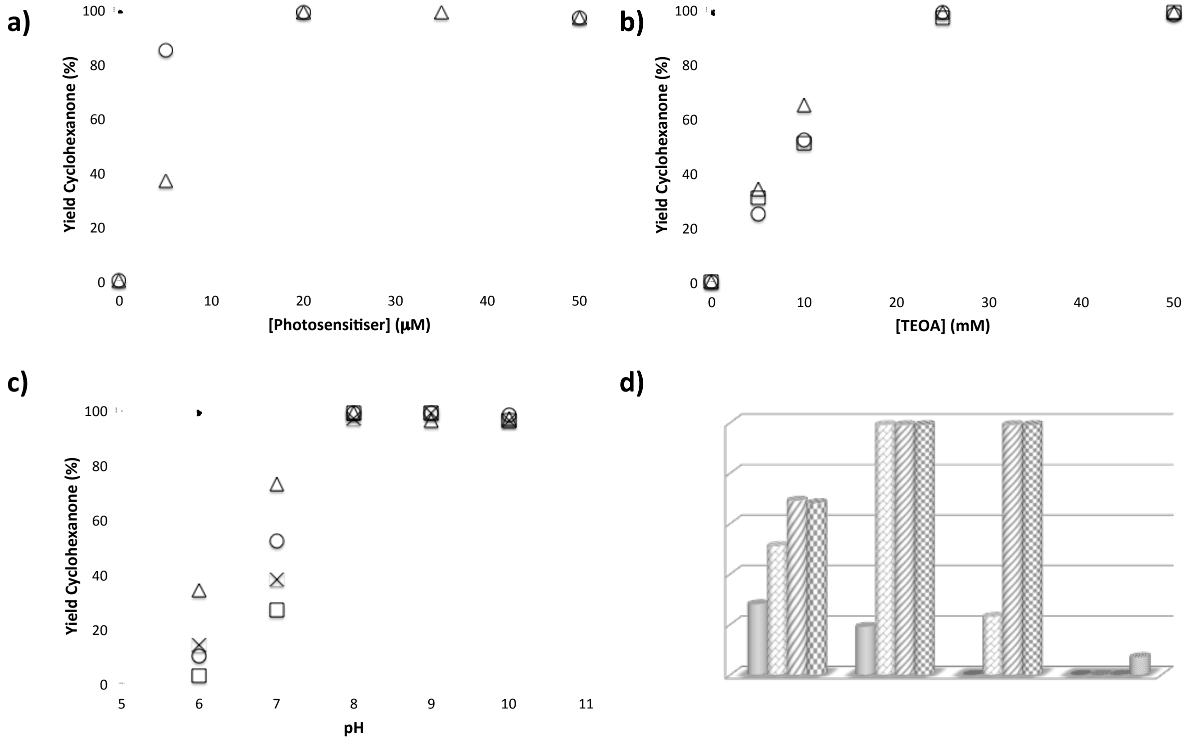

Fig. S5. Summary of the optimisation studies of the photosensitizer-driven OYE-catalysed reduction of cyclohexen-2-one to cyclohexanone. a) Effect of the photosensitizer concentration in the presence of excess MV^{2+} and TEA. Data for TOYE and PETNR is shown as open circles and triangles, respectively. b) Reaction dependence on the concentration of the sacrificial electron donor TEA with three photosensitizers. Data for OYE-photosensitizer: TOYE-$[Ru(bpz)_2(dClbpy)]^{2+}$, PETNR-$[Ru(bpz)_2(dClbpy)]^{2+}$ and PETNR-$[Ir(Me\text{-}2,2'\text{-}bpy^{+})_2(bpy)]^{3+}$ are shown as open circles, rectangles and squares, respectively. c) pH optimisation studies of TOYE and $PETNR_{R324C}$ with two photosensitizers. Data for OYE-photosensitizer: TOYE-$[Ru(bpz)_2(dClbpy)]Cl_2$, TOYE- $[Ir(Me\text{-}2,2'\text{-}bpy)_2(bpy)]Cl_3$, $PETNR_{R3254C}$-$[Ru(bpz)_2(dClbpy)]Cl_2$ and $PETNR_{R3254C}$-$[Ir(Me\text{-}2,2'\text{-}bpy)_2(bpy)]Cl_3$ are shown as open circles, rectangles, squares and crosses, respectively. d) Effect of the photosensitising wavelength on $PETNR_{R3254C}$ activity with four photosensitizers. Data for wavelengths 530, 460, 360 and 305 nm are shown as solid, brick-effect, diagonal stripes and diamond cylinders, respectively; PS1 = $[Ru(bpy)(Me_2qpy^{2+})_2]Cl_6$, PS2 = $[Ru(bpz)_2(dClbpy)]Cl_2$, PS3 = $[Ir(Me\text{-}2,2'\text{-}bpy)_2(bpy)]Cl_3$, PS4 = $[Ir(Me\text{-}3,2'\text{-}bpy)_2(dCF_3bpy)]Cl_3$.

S2.3 Effect of the sacrificial electron donor concentration on OYE activity

The yields of cyclohexanone produced by OYEs using either $[Ru(bpz)_2(dClbpy)]^{2+}$ or $[Ir(Me\text{-}2,2'\text{-}bpy)_2(bpy)]^{3+}$ depend on the concentration of the sacrificial electron donor TEA (Table S3 and Figure S6). However, in each case, maximal product yields are obtained with 25 mM TEA (Figure S5b). These results are consistent with the proposed mechanism where turnover is limited by the rate of generation of the reduced sensitizer upon quenching of the excited state by TEA (Scheme 2a). This is in contrast to direct light-driven flavin reduction mechanisms of PAMO-P3 and YqjM, which both exhibit initial rates independent of the donor concentration.[2,3]

Table S3 Influence of changes in sacrificial electron donor concentrations upon the rates of light-driven biocatalytic reduction of cyclohexen-2-one by PETNR or TOYE.

[TEA] [mM]	Enzyme	Photosensitizer	TOF [a]	Conv.[b] [%]	Yield[b] [%]
5	TOYE	$[Ru(bpz)_2(dClbpy)]^{2+}$	45	28	25
	PETNR	$[Ru(bpz)_2(dClbpy)]^{2+}$	45	36	34
	PETNR	$[Ir(Me\text{-}2,2'\text{-}bpy)_2(bpy)]^{3+}$	60	37	31
10	TOYE	$[Ru(bpz)_2(dClbpy)]^{2+}$	80	52	52
	PETNR	$[Ru(bpz)_2(dClbpy)]^{2+}$	80	70	65
	PETNR	$[Ir(Me\text{-}2,2'\text{-}bpy)_2(bpy)]^{3+}$	75	51	51
25	TOYE	$[Ru(bpz)_2(dClbpy)]^{2+}$	120	100	>99
	PETNR	$[Ru(bpz)_2(dClbpy)]^{2+}$	125	100	>99
	PETNR	$[Ir(Me\text{-}2,2'\text{-}bpy)_2(bpy)]^{3+}$	100	100	97
50	TOYE	$[Ru(bpz)_2(dClbpy)]^{2+}$	210	100	98
	PETNR	$[Ru(bpz)_2(dClbpy)]^{2+}$	230	100	>99
	PETNR	$[Ir(Me\text{-}2,2'\text{-}bpy)_2(bpy)]^{3+}$	190	100	>99

[a] Determined after 120 min. [b]Determined by GC analysis after 240 min, except reactions indicated by * which were analysed after 60 min.

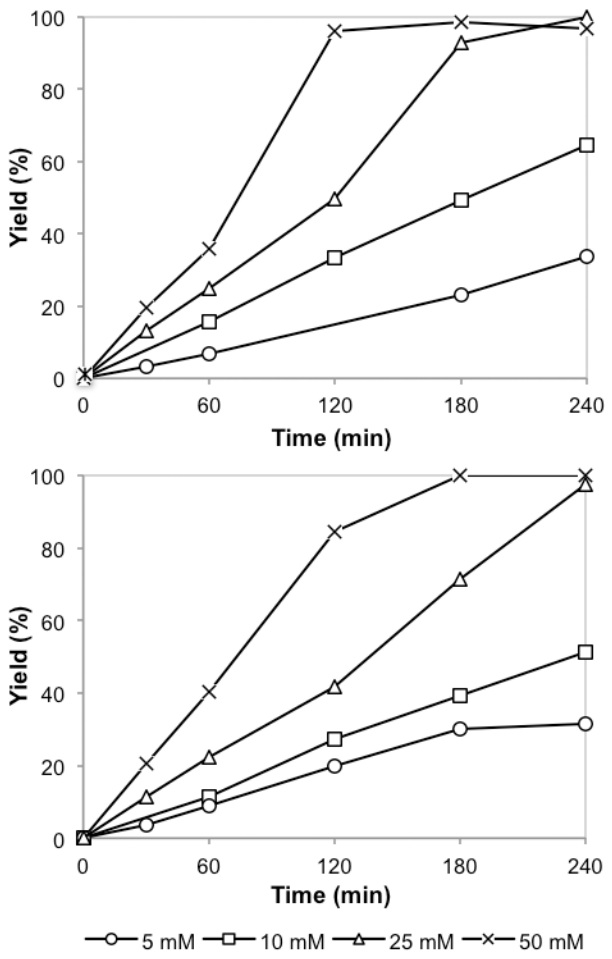

Fig. S6 Influence of TEA concentration upon the light-driven bioreduction of cyclohexen-2-one by $PETNR_{R324C}$ using $[Ru(bpz)_2(dClbpy)]Cl_2$ (top) or $[Ir(1\text{-}Me\text{-}2,2'\text{-}bpy)_2(bpy)]Cl_3$ (bottom) as photosensitizer.

S2.4 pH dependence of OYE activity

Table S4 The pH dependence of the light-driven biocatalytic reduction of cyclohexen-2-one by PETNR or TOYE.

pH	Enzyme	Photosensitizer	TOF [a]	Conv.[b] [%]	Yield[b] [%]
6	TOYE	$[Ru(bpz)_2(dClbpy)]Cl_2$	10	10	10
	TOYE	$[Ir(Me\text{-}2,2'\text{-}bpy)_2(bpy)]Cl_3$	50	38	34
	$PETNR_{R324C}$	$[Ru(bpz)_2(dClbpy)]Cl_2$	5	4	3
	$PETNR_{R324C}$	$[Ir(Me\text{-}2,2'\text{-}bpy)_2(bpy)]Cl_3$	15	16	14
7	TOYE	$[Ru(bpz)_2(dClbpy)]Cl_2$	55	54	52
	TOYE	$[Ir(Me\text{-}2,2'\text{-}bpy)_2(bpy)]Cl_3$	70	74	73
	$PETNR_{R324C}$	$[Ru(bpz)_2(dClbpy)]Cl_2$	30	27	27
	$PETNR_{R324C}$	$[Ir(Me\text{-}2,2'\text{-}bpy)_2(bpy)]Cl_3$	40	39	38
8	TOYE	$[Ru(bpz)_2(dClbpy)]Cl_2$	130	100	>99
	TOYE	$[Ir(Me\text{-}2,2'\text{-}bpy)_2(bpy)]Cl_3$	125	100	>99
	$PETNR_{R324C}$	$[Ru(bpz)_2(dClbpy)]Cl_2$	125	100	>99
	$PETNR_{R324C}$	$[Ir(Me\text{-}2,2'\text{-}bpy)_2(bpy)]Cl_3$	100	100	97
9	TOYE	$[Ru(bpz)_2(dClbpy)]Cl_2$	250	100	>99
	TOYE	$[Ir(Me\text{-}2,2'\text{-}bpy)_2(bpy)]Cl_3$	155	100	96
	$PETNR_{R324C}$	$[Ru(bpz)_2(dClbpy)]Cl_2$	275	100	>99
	$PETNR_{R324C}$	$[Ir(Me\text{-}2,2'\text{-}bpy)_2(bpy)]Cl_3$	140	100	>99
10	TOYE	$[Ru(bpz)_2(dClbpy)]Cl_2$	290	100	98
	TOYE	$[Ir(Me\text{-}2,2'\text{-}bpy)_2(bpy)]Cl_3$	145	100	97
	$PETNR_{R324C}$	$[Ru(bpz)_2(dClbpy)]Cl_2$	210	100	96
	$PETNR_{R324C}$	$[Ir(Me\text{-}2,2'\text{-}bpy)_2(bpy)]Cl_3$	180	100	96

[a] Determined after 120 min. [b]Determined by GC analysis after 240 min, except reactions indicated by * which were analysed after 60 min.

The pH dependence of the reaction was determined in TEA buffer solutions (pH 6–10) using two photosensitizers ($[Ru(bpz)_2(dClbpy)]Cl_2$ or $Ir(Me\text{-}2,2'\text{-}bpy)_2(bpy)]Cl_3$) and TOYE or $PETNR_{R324C}$ (Table S4 and Figure S7). In all instances, maximal product yield is obtained in the pH range 8–10 (Figure S5c). However the TOFs suggest the optimum pH values lie between 9 and 10 (Table S4). These results are consistent with prior studies showing that NAD(P)H-mediated reactions of PETNR have

a broad pH activity profile, with lower conversion rates observed at pH < 7.[4] In the present system, if enzyme deactivation were the sole contributor to the poor performance at low pH, an accumulation of $MV^{+\bullet}$ would be expected due to perturbation of oxidative quenching by FMN. However, no significant concentration of $MV^{+\bullet}$ was detected during reactions at pH 6–7. Therefore, the pH dependency may in part be attributed to protonation of TEA. The TEA cation, formed on reductive quenching of the excited complex, is also subject to an acid-base equilibrium in solution. At low pH, the cationic form persists in solution and may act as an oxidant towards $MV^{+\bullet}$, thus further perturbing the forward electron transfer within the catalytic cycle.[5] Similar observations and rationale have been applied to systems employing EDTA as a sacrificial donor.[6,7]

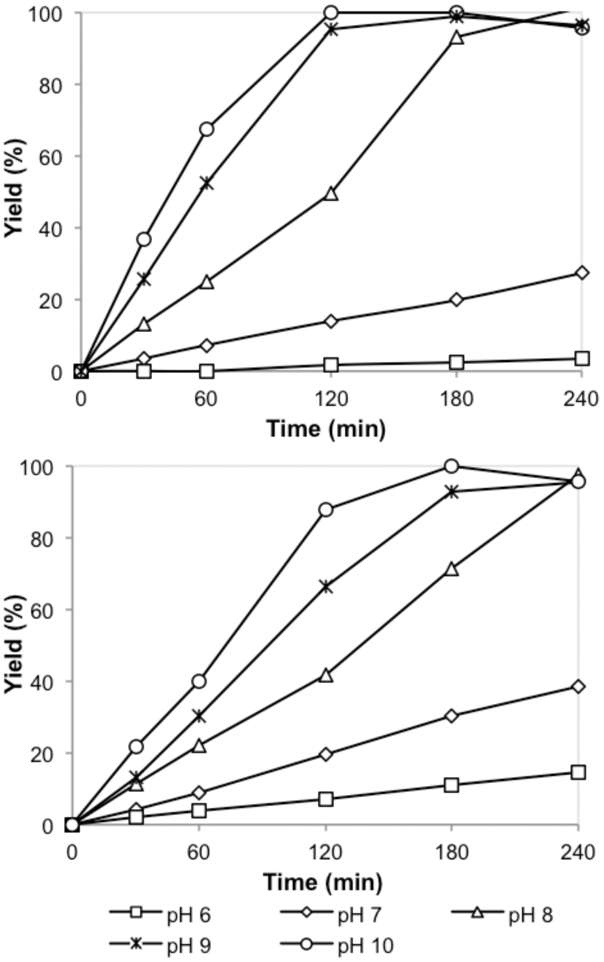

Fig. S7 Influence of pH on the light-driven $PETNR_{R324C}$-catalyzed reduction of 2-cyclohexenone using $[Ru(bpz)_2(dClbpy)]Cl_2$ (top) or $[Ir(1\text{-}Me\text{-}2,2'\text{-}bpy)_2(bpy)]Cl_3$ (bottom) as photosensitizer.

The $[Ru(bpz)_2(L–L)]^{2+}$ complexes show a greater pH dependence than the Ir(III) compounds, with a dramatic decrease in activity with increasing acidity of the buffer. This can be ascribed to the deactivation of the photosensitizer upon protonation of the uncoordinated N atoms of the bpz ligands. The ligand-centred radical that is formed upon reductive quenching of the excited sensitizer may be readily protonated at sufficiently low pH to form the conjugate acid $[Ru(bpz)_2(\bullet bpzH)]^{2+}$, which has $pK_a \approx 7.1$.[8] As a consequence, the redox potentials of the complex undergo an anodic shift of *ca.* 0.2 V, becoming insufficient to reduce MV^{2+}.[8] This explains the near inactivity of the system at pH 6, as at this point the majority of the complex is expected to be protonated.

S2.5 Effect of the irradiation wavelength on OYE activity

The varying absorption profiles and redox properties of transition metal complexes suggest that different photosensitizers may be subject to selective excitation at specific wavelengths, enabling a finer level of control over their reactions. Therefore, photosensitizers displaying the greatest diversity in absorption profiles ([Ru(bpy)(Me$_2$qpy^{2+})$_2$]Cl$_6$, [Ru(bpz)$_2$(dClbpy)]Cl$_2$, [Ir(Me-2,2′-bpy)$_2$(bpy)]Cl$_3$ and [Ir(Me-3,2′-bpy)$_2$(dCF$_3$bpy)]Cl$_3$; Figure S8) underwent biotransformations with PETNR$_{R324C}$, using specific wavelengths for photoexcitation (Table S5 and Figure S9). Experiments utilised a series of long pass optical filters (530, 460, 360 and 305 nm), which attenuate light of higher energy and allow for selective excitation of transitions that occur at longer wavelengths.

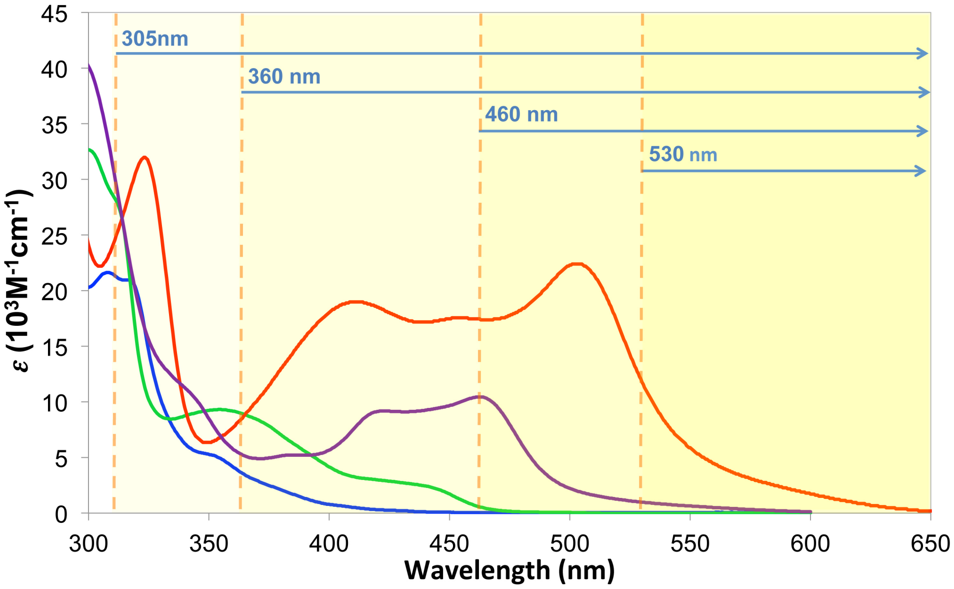

Fig. S8 UV-Vis absorption spectra of [Ru(bpy)(Me$_2$qpy^{2+})$_2$]Cl$_6$ (red), [Ru(bpz)$_2$(dClbpy)]Cl$_2$ (purple), [Ir(Me-2,2′-bpy)$_2$(bpy)]Cl$_3$ (green) and [Ir(Me-3,2′-bpy)$_2$(dCF$_3$bpy)]Cl$_3$ (blue) recorded in deionized water; highlighting indicates the long pass optical filters used for selective photoexcitation.

Absorption by the low intensity tailing of the MLCT band in [Ru(bpz)$_2$(dClbpy)]Cl$_2$ is sufficient to enable minor product formation by PETNR$_{R324C}$ on irradiation with light of $\lambda \geq 530$ nm (19% yield after 4 h; Figure S5d, Table S5). In contrast, no substrate turnover is observed with either [Ir(Me-2,2′-bpy)$_2$(bpy)]Cl$_3$ or [Ir(Me-3,2′-bpy)$_2$(dCF$_3$bpy)]Cl$_3$ with such long wavelength excitation. Of the four sensitizers, [Ru(bpy)(Me$_2$qpy^{2+})$_2$]Cl$_6$ is the most effective, although the difference in activity compared to [Ru(bpz)$_2$(dClbpy)]Cl$_2$ does not follow the changes in absorption intensity. On illumination at $\lambda \geq 460$ nm, PETNR$_{R324C}$ activity increases significantly with both [Ru(bpy)(Me$_2$qpy^{2+})$_2$]Cl$_6$ and [Ru(bpz)$_2$(dClbpy)]Cl$_2$ (Figure S5d, Table S5), especially the latter, despite its relatively weaker MLCT bands. Some PETNR$_{R324C}$ activity is seen with [Ir(Me-2,2′-bpy)$_2$(bpy)]Cl$_3$ at $\lambda \geq 460$ nm, exhibiting rates comparable to [Ru(bpz)$_2$(dClbpy)]Cl$_2$ at $\lambda \geq 530$ nm. By using a long-pass filter of *ca.* $\lambda \geq 500$ nm, it may be possible to achieve high activity with the Ru(II) complexes without initiating turnover with [Ir(Me-2,2′-bpy)$_2$(bpy)]Cl$_3$. Hence, truly selective activation in a system comprising multiple sensitizers may be a realistic goal. At $\lambda \geq 305$ nm, catalytic turnover using [Ir(Me-3,2′-bpy)$_2$(dCF$_3$bpy)]Cl$_3$ as a sensitizer becomes detectable (Figure S5d), indicating unsuitability for use as a general OYE photosensitizer. It is clear that sufficiently

high levels of $PETNR_{R324C}$ activity may be achieved with these Ru(II) photosensitizers on excitation of the lower energy MLCT band only. Using low energy light is beneficial in terms of avoiding irreversible photochemical damage, thus enhancing the longevity of a catalytic system.

Table S5 Influence of the wavelength of incident irradiation in the light-driven bioreduction of cyclohexen-2-one by $PETNR_{R324C}$.

Photosensitizer	λ_{filter} (nm)	TOF [a]	Conv.[b] [%]	Yield[b] [%]
$[Ru(bpy)(Me_2qpy^{2+})_2]Cl_6$	530	40	30	28
	460	70	51	51
	360	90	76	69
	305	78.7	68	68
$[Ru(bpz)_2(dClbpy)]Cl_2$	530	27.0	20	19
	460	133.8	100	>99
	360	123.6	100	>99
	305	188.2	100	>99
$[Ir(Me\text{-}2,2'\text{-}bpy)_2(bpy)]Cl_3$	530	-	0	0
	460	28.1	23	23
	360	129.1	100	>99
	305	100.0	100	>99
$[Ir(Me\text{-}3,2'\text{-}bpy)_2(dCF_3bpy)]Cl_3$	530	-	0	0
	460	-	0	0
	360	-	0	0
	305	8.7	8	7

[a] Determined after 120 min. [b]Determined by GC analysis after 240 min, except reactions indicated by * which were analysed after 60 min.

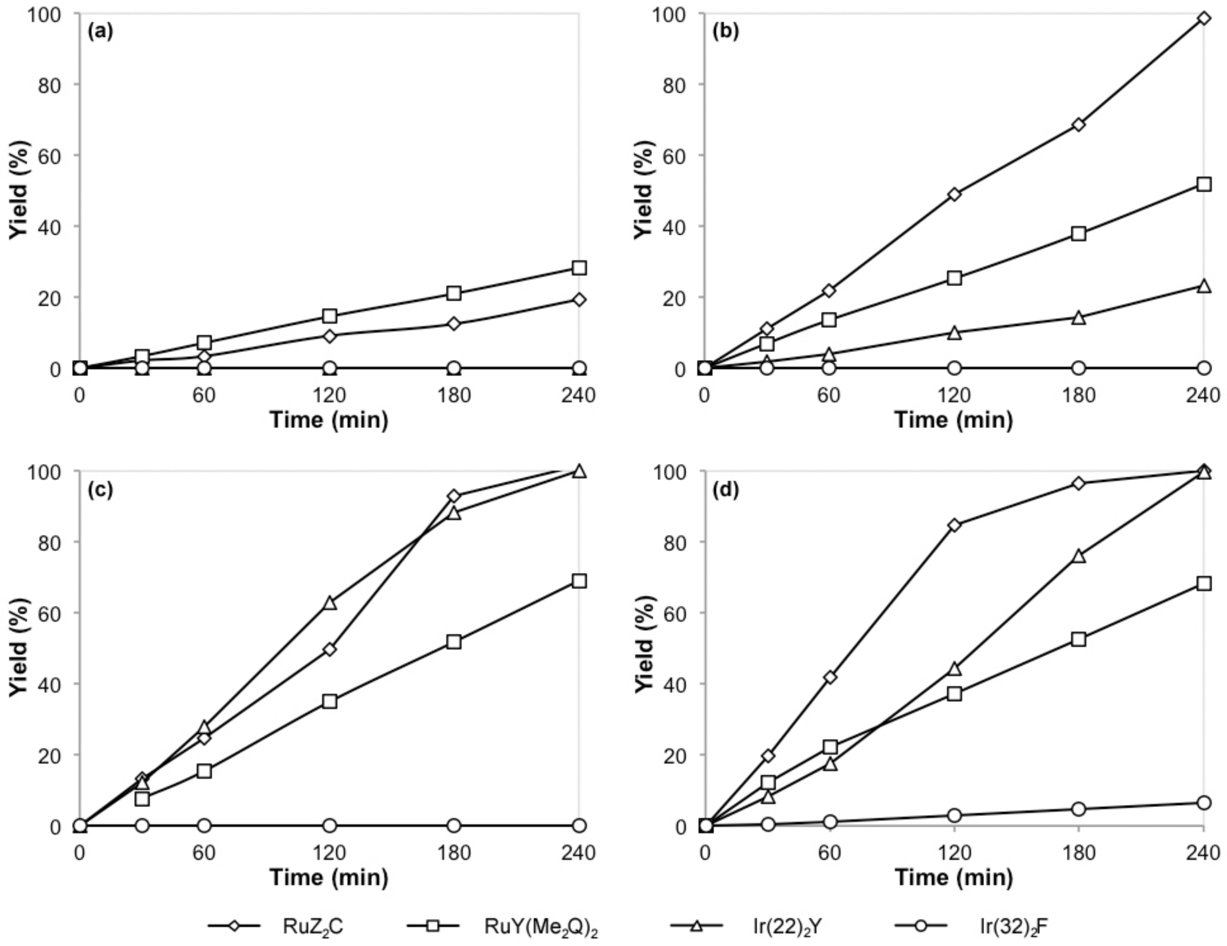

Fig. S9 Influence of the photosensitizer and the wavelength of incident irradiation upon the light-driven bioreduction of cyclohexen-2-one by $PETNR_{R324C}$, as demonstrated by the use of long-pass filters at (a) 530; (b) 460; (c) 360 and (d) 305 nm.

S2.6 Biotransformation data

Table S6 Biphasic reduction of various activated alkenes by PETNR and TOYE using a photosensitizer or a NADP$^+$/G6PDH co-factor regeneration system.[a]

Substrate	Catalyst[a]	Solvent	Conv.[b] [%]	Yield[b] [%]	*ee*[b] %
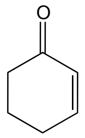	TOYE/Ru	*n*-octanol	100	>98	--
	TOYE/Ru	isooctane	100	>98	--
	TOYE/Ru	TBME	100	>98	--
	PETNR$_{R324C}$/Ru	*n*-octanol	100	>98	--
	PETNR$_{R324C}$/Ru	isooctane	100	>98	--
	PETNR$_{R324C}$/Ru	TBME	100	>98	--
	TOYE/G6PDH	*n*-octanol	100	>98	--
	TOYE/G6PDH	isooctane	100	>98	--
	TOYE/G6PDH	TBME	100	>98	--
	PETNR$_{R324C}$ /G6PDH	*n*-octanol	100	>98	--
	PETNR$_{R324C}$ /G6PDH	isooctane	100	>98	--
	PETNR$_{R324C}$ /G6PDH	TBME	24	14	--
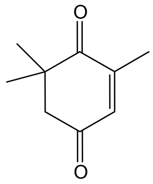	TOYE/Ru	*n*-octanol	100	87	20 (*R*)
	TOYE/Ru	isooctane	100	89	26 (*R*)
	TOYE/Ru	TBME	100	75	23 (*R*)
	PETNR$_{R324C}$/Ru	*n*-octanol	100	84	65 (*R*)
	PETNR$_{R324C}$/Ru	isooctane	100	97	26 (*R*)
	PETNR$_{R324C}$/Ru	TBME	100	87	29 (*R*)
	TOYE/G6PDH	*n*-octanol	100	90	67 (*R*)
	TOYE/G6PDH	isooctane	100	92	43 (*R*)
	TOYE/G6PDH	TBME	100	87	71 (*R*)
	PETNR$_{R324C}$ /G6PDH	*n*-octanol	100	86	66 (*R*)
	PETNR$_{R324C}$ /G6PDH	isooctane	100	92	43 (*R*)
	PETNR$_{R324C}$ /G6PDH	TBME	53	44	73 (*R*)
	TOYE/Ru	*n*-octanol	90	86	--
	TOYE/Ru	isooctane	100	>98	--
	TOYE/Ru	TBME	98	90	--

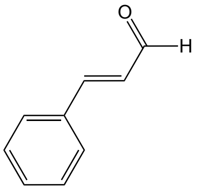	$PETNR_{R324C}$/Ru	*n*-octanol	100	96	--
	$PETNR_{R324C}$/Ru	isooctane	100	>98	--
	$PETNR_{R324C}$/Ru	TBME	100	>98	--
	TOYE/G6PDH	*n*-octanol	82	80	--
	TOYE/G6PDH	isooctane	100	>98	--
	TOYE/G6PDH	TBME	68	67	--
	$PETNR_{R324C}$ /G6PDH	*n*-octanol	100	96	--
	$PETNR_{R324C}$ /G6PDH	isooctane	100	97	--
	$PETNR_{R324C}$ /G6PDH	TBME	55	50	--
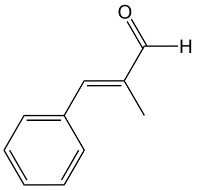	TOYE/Ru	*n*-octanol	100	>98	15 (*S*)
	TOYE/Ru	isooctane	100	91	7 (*S*)
	TOYE/Ru	TBME	100	>98	13 (*S*)
	$PETNR_{R324C}$/Ru	*n*-octanol	100	>98	23 (*S*)
	$PETNR_{R324C}$/Ru	isooctane	100	95	6 (*S*)
	$PETNR_{R324C}$/Ru	TBME	100	>98	*rac*
	TOYE/G6PDH	*n*-octanol	76	76	22 (*S*)
	TOYE/G6PDH	isooctane	100	96	16 (*S*)
	TOYE/G6PDH	TBME	77	76	4 (*S*)
	$PETNR_{R324C}$ /G6PDH	*n*-octanol	100	98	13 (*S*)
	$PETNR_{R324C}$ /G6PDH	isooctane	100	95	12 (*S*)
	$PETNR_{R324C}$ /G6PDH	TBME	57	56	8 (*S*)
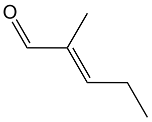	TOYE/Ru	*n*-octanol	100	85	30 (*S*)
	TOYE/Ru	isooctane	100	>98	14 (*S*)
	TOYE/Ru	TBME	100	>98	8 (*S*)
	$PETNR_{R324C}$/Ru	*n*-octanol	100	>98	90 (*S*)
	$PETNR_{R324C}$/Ru	isooctane	100	93	89 (*S*)
	$PETNR_{R324C}$/Ru	TBME	100	>98	75 (*S*)
	TOYE/G6PDH	*n*-octanol	100	>98	30 (*S*)
	TOYE/G6PDH	isooctane	100	98	17 (*S*)
	TOYE/G6PDH	TBME	100	>98	7 (*S*)
	$PETNR_{R324C}$	*n*-octanol	100	>98	87 (*S*)

	/G6PDH				
	PETNR$_{R324C}$ /G6PDH	isooctane	100	96	84 (*S*)
	PETNR$_{R324C}$ /G6PDH	TBME	100	>98	81 (*S*)
	TOYE/Ru	*n*-octanol	100	54	>99 (*R*)
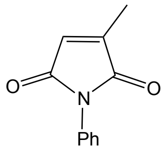	TOYE/Ru	isooctane	--	--	--
	TOYE/Ru	TBME	100	67	>99 (*R*)
	PETNR$_{R324C}$/Ru	*n*-octanol	100	47	>99 (*R*)
	PETNR$_{R324C}$/Ru	isooctane	--	--	--
	PETNR$_{R324C}$/Ru	TBME	81	28	>99 (*R*)
	TOYE/G6PDH	*n*-octanol	100	89	>99 (*R*)
	TOYE/G6PDH	isooctane	--	--	--
	TOYE/G6PDH	TBME	100	66	>99 (*R*)
	PETNR$_{R324C}$ /G6PDH	*n*-octanol	100	81	>99 (*R*)
	PETNR$_{R324C}$ /G6PDH	isooctane	--	--	--
	PETNR$_{R324C}$ /G6PDH	TBME	78	38	>99 (*R*)
	TOYE/Ru	*n*-octanol	94	90	97 (2*R*,5*S*)
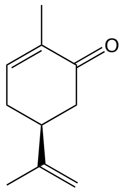	TOYE/Ru	isooctane	90	86	97 (2*R*,5*S*)
	TOYE/Ru	TBME	98	95	97 (2*R*,5*S*)
	PETNR$_{R324C}$/Ru	*n*-octanol	100	97	97 (2*R*,5*S*)
	PETNR$_{R324C}$/Ru	isooctane	100	99	96 (2*R*,5*S*)
	PETNR$_{R324C}$/Ru	TBME	92	90	93 (2*R*,5*S*)
	TOYE/G6PDH	*n*-octanol	97	92	97 (2*R*,5*S*)
	TOYE/G6PDH	isooctane	100	98	96 (2*R*,5*S*)
	TOYE/G6PDH	TBME	100	96	97 (2*R*,5*S*)
	PETNR$_{R324C}$ /G6PDH	*n*-octanol	98	97	96 (2*R*,5*S*)
	PETNR$_{R324C}$ /G6PDH	isooctane	99	84	96 (2*R*,5*S*)

$PETNR_{R324C}$ /G6PDH	TBME	47	46	98 (2*R*,5*S*)

[a] Conditions for assays containing a photosensitizer: Enzyme (10 μM), $[Ru(bpz)_2(dClbpy)]Cl_2$ (20 μM) and $[MV^{2+}]Cl_2$ (0.1 mM) in TEA buffer (1.0 mL, 50 mM, pH 8.0). Conditions for assays containing G6PDH regeneration system: Enzyme (10 μM), $NADP^+$ (10 μM), glucose-6-phosphate (15 mM) and glucose-6-phosphate dehydrogenase (G6PDH, 10 units) in phosphate buffer (1.0 mL, 50 mM, pH 8.0). Substrate added as a solution in the indicated solvent (25 mM, 200 μL). Assays undertaken at RT for 24 h at 450 rpm. [b]Conversions, yields and product *ee* determined by GC analysis calculated after 24 h.

References

1. L. L. C. Schrodinger, 2010.
2. M. M. Grau, J. C. van der Toorn, L. G. Otten, P. Macheroux, A. Taglieber, F. E. Zilly, I. W. C. E. Arends and F. Hollman, *Adv Synth Catal*, 2009, **351**, 3279.
3. A. Taglieber, F. Schulz, F. Hollman, M. Rusek and M. T. Reetz, *ChemBioChem*, 2008, **9**, 565.
4. A. Fryszkowska, H. S. Toogood, M. Sakuma, J. M. Gardiner, G. M. Stephens and N. S. Scrutton, *Adv Synth Catal*, 2009, **351**, 2976.
5. K. Kalyanasundaram, J. Kiwi and M. Gratzel, *Helv Chim Acta*, 1978, **61**, 2720.
6. A. Harriman and A. Mills, *J Chem Soc Faraday Trans 2 Mol Chem Phys*, 1981, **77**, 2111.
7. D. Miller and G. McLendon, *Inorg Chem*, 1981, **20**, 950.
8. M. Venturi, Q. G. Mulazzani, M. Ciano and M. Z. Hoffman, *Inorg Chem*, 1986, **25**, 4493.
